# Supplementary material for: Prevalence of Depression and Anxiety in Nurses during the First Eleven Months of the COVID-19 Pandemic: A Systematic Review and Meta-Analysis
Source: Int J Environ Res Public Health. 2022 Jan 20;19(3):1154. doi: 10.3390/ijerph19031154 (PMC8834441; doi:10.3390/ijerph19031154)
Supplement: Supplementary file 1 [file ijerph-19-01154-s001.zip › ijerph-1528794-supplementary.pdf]

International Journal of
Environmental Research and Public Health

MDPI

Supplementary Materials

Incidence of Depression and Anxiety in Nurses During the First 11 Months of the COVID-19 Pandemic: A Systematic Review and Meta-Analysis

Barbara Ślusarska, Grzegorz Józef Nowicki, Barbara Niedorys-Karczmarczyk and Agnieszka Chrzan-Rodak

Table S1. PRISMA Checklist.

Section/topic	#	Checklist items	Reported on page #
**TITLE**			
Title	1	Identify the report as a systematic review, meta-analysis, or both.	page 1
**ABSTRACT**			
Structured summary	2	Provide a structured summary including, as applicable: background; objectives; data sources; study eligibility criteria, participants, and interventions; study appraisal and synthesis methods; results; limitations; conclusions and implications of key findings; systematic review registration number.	page 1
**INTRODUCTION**			
Rationale	3	Describe the rationale for the review in the context of what is already known.	page 2
Objectives	4	Provide an explicit statement of questions being addressed with reference to participants, interventions, comparisons, outcomes, and study design (PICOS).	page 3
**METHODS**			
Protocol and registration	5	Indicate if a review protocol exists, if and where it can be accessed (e.g., Web address), and, if available, provide registration information including registration number.	page 4
Eligibility criteria	6	Specify study characteristics (e.g., PICOS, length of follow-up) and report characteristics (e.g., years considered, language, publication status) used as criteria for eligibility, giving rationale.	pp.4-5
Information sources	7	Describe all information sources (e.g., databases with dates of coverage, contact with study authors to identify additional studies) in the search and date last searched.	page 4-5
Search	8	Present full electronic search strategy for at least one database, including any limits used, such that it could be repeated.	page 4
Study selection	9	State the process for selecting studies (i.e., screening, eligibility, included in systematic review, and, if applicable, included in the meta-analysis).	page 4-5
Data collection process	10	Describe method of data extraction from reports (e.g., piloted forms, independently, in duplicate) and any processes for obtaining and	pp.4-5

		confirming data from investigators.	
Data items	11	List and define all variables for which data were sought (e.g., PICOS, funding sources) and any assumptions and simplifications made.	page 5
Risk of bias in individual studies	12	Describe methods used for assessing risk of bias of individual studies (including specification of whether this was done at the study or outcome level), and how this information is to be used in any data synthesis.	page 5
Summary measures	13	State the principal summary measures (e.g., risk ratio, difference in means).	page 5
Synthesis of results	14	Describe the methods of handling data and combining results of studies, if done, including measures of consistency (e.g., I^2) for each meta-analysis.	page 6
Risk of bias across studies	15	Specify any assessment of risk of bias that may affect the cumulative evidence (e.g., publication bias, selective reporting within studies).	page 10
Additional analyses	16	Describe methods of additional analyses (e.g., sensitivity or subgroup analyses, meta-regression), if done, indicating which were pre-specified.	-
**RESULTS**			
Study selection	17	Give numbers of studies screened, assessed for eligibility, and included in the review, with reasons for exclusions at each stage, ideally with a flow diagram.	page 6-7
Study characteristics	18	For each study, present characteristics for which data were extracted (e.g., study size, PICOS, follow-up period) and provide the citations.	page 6-7
Risk of bias within studies	19	Present data on risk of bias of each study and, if available, any outcome level assessment (see item 12).	page 10
Results of individual studies	20	For all outcomes considered (benefits or harms), present, for each study: (a) simple summary data for each intervention group (b) effect estimates and confidence intervals, ideally with a forest plot.	pp. 9-10
Synthesis of results	21	Present results of each meta-analysis done, including confidence intervals and measures of consistency.	pp. 8-10
Risk of bias across studies	22	Present results of any assessment of risk of bias across studies (see Item 15).	page 10
Additional analysis	23	Give results of additional analyses, if done (e.g., sensitivity or subgroup analyses, meta-regression [see Item 16]).	-
**DISCUSSION**			
Summary of evidence	24	Summarize the main findings including the strength of evidence for each main outcome; consider their relevance to key groups (e.g., healthcare providers, users, and policy makers).	pp.10-12
Limitations	25	Discuss limitations at study and outcomelevel (e.g., risk of bias), and at review-level (e.g., incomplete retrieval of identified research, reporting bias).	pp.14-15
Conclusions	26	Provide a general interpretation of the results in the context of other evidence, and implications for future research.	page 15
**FUNDING**			
Funding	27	Describe sources of funding for the systematic review and other support	Title page

(e.g., supply of data); role of funders for the systematic review.

Table S2. Search strategy and search terms.

Database	Search terms
**PubMed**	#1 ((COVID OR Covid OR COVID-19 OR COVID 19 OR COVID19 OR severe acute respiratory syndrome OR coronavirus 2 OR SARS-CoV-2 OR SARSCoV2 OR SARS CoV 2 OR 2019-nCoV OR 2019 Novel Coronavirus OR Coronavirus Disease 2019 OR Coronavirus Disease-19 OR SARS Coronavirus 2) AND (mental health)) AND (Nurse OR Nurses OR Nursing Personnel OR Registered Nurses OR Nursing Staff)<br>#2 ((COVID OR Covid OR COVID-19 OR COVID 19 OR COVID19 OR severe acute respiratory syndrome OR coronavirus 2 OR SARS-CoV-2 OR SARSCoV2 OR SARS CoV 2 OR 2019-nCoV OR 2019 Novel Coronavirus OR Coronavirus Disease 2019 OR Coronavirus Disease-19 OR SARS Coronavirus 2) AND (mental wellbeing)) AND (Nurse OR Nurses OR Nursing Personnel OR Registered Nurses OR Nursing Staff)<br>#3 ((COVID OR Covid OR COVID-19 OR COVID 19 OR COVID19 OR severe acute respiratory syndrome OR coronavirus 2 OR SARS-CoV-2 OR SARSCoV2 OR SARS CoV 2 OR 2019-nCoV OR 2019 Novel Coronavirus OR Coronavirus Disease 2019 OR Coronavirus Disease-19 OR SARS Coronavirus 2) AND (mental health disorders)) AND (Nurse OR Nurses OR Nursing Personnel OR Registered Nurses OR Nursing Staff)<br>#4 ((COVID OR Covid OR COVID-19 OR COVID 19 OR COVID19 OR severe acute respiratory syndrome OR coronavirus 2 OR SARS-CoV-2 OR SARSCoV2 OR SARS CoV 2 OR 2019-nCoV OR 2019 Novel Coronavirus OR Coronavirus Disease 2019 OR Coronavirus Disease-19 OR SARS Coronavirus 2) AND (depression)) AND (Nurse OR Nurses OR Nursing Personnel OR Registered Nurses OR Nursing Staff)<br>#5 ((COVID OR Covid OR COVID-19 OR COVID 19 OR COVID19 OR severe acute respiratory syndrome OR coronavirus 2 OR SARS-CoV-2 OR SARSCoV2 OR SARS CoV 2 OR 2019-nCoV OR 2019 Novel Coronavirus OR Coronavirus Disease 2019 OR Coronavirus Disease-19 OR SARS Coronavirus 2) AND (anxiety)) AND (Nurse OR Nurses OR Nursing Personnel OR Registered Nurses OR Nursing Staff)<br>#6 ((COVID OR Covid OR COVID-19 OR COVID 19 OR COVID19 OR severe acute respiratory syndrome OR coronavirus 2 OR SARS-CoV-2 OR SARSCoV2 OR SARS CoV 2 OR 2019-nCoV OR 2019 Novel Coronavirus OR Coronavirus Disease 2019 OR Coronavirus Disease-19 OR SARS Coronavirus 2) AND (stress disorder OR stress)) AND (Nurse OR Nurses OR Nursing Personnel OR Registered Nurses OR Nursing Staff)<br>#7 ((COVID OR Covid OR COVID-19 OR COVID 19 OR COVID19 OR severe acute respiratory syndrome OR coronavirus 2 OR SARS-CoV-2 OR SARSCoV2 OR SARS CoV 2 OR 2019-nCoV OR 2019 Novel Coronavirus OR Coronavirus Disease 2019 OR Coronavirus Disease-19 OR SARS Coronavirus 2) AND (psychological distress)) AND (Nurse OR Nurses OR Nursing Personnel OR Registered Nurses OR Nursing Staff)<br>#8 ((COVID OR Covid OR COVID-19 OR COVID 19 OR COVID19 OR severe acute respiratory syndrome OR coronavirus 2 OR SARS-CoV-2 OR SARSCoV2 OR SARS CoV 2 OR 2019-nCoV OR 2019 Novel Coronavirus OR Coronavirus Disease 2019 OR Coronavirus Disease-19 OR SARS Coronavirus 2)

	AND (post-traumatic stress OR PTSD)) AND (Nurse OR Nurses OR Nursing Personnel OR Registered Nurses OR Nursing Staff)
**Web of Science**	#1 TOPIC: (COVID OR Covid OR COVID-19 OR COVID 19 OR COVID19 OR severe acute respiratory syndrome OR coronavirus 2 OR SARS-CoV-2 OR SARSCoV2 OR SARS CoV 2 OR 2019-nCoV OR 2019 Novel Coronavirus OR Coronavirus Disease 2019 OR Coronavirus Disease-19 OR SARS Coronavirus 2) AND TOPIC: (mental health) AND TOPIC: (Nurse OR Nurses OR Nursing Personnel OR Registered Nurses OR Nursing Staff)<br>#2 TOPIC: (COVID OR Covid OR COVID-19 OR COVID 19 OR COVID19 OR severe acute respiratory syndrome OR coronavirus 2 OR SARS-CoV-2 OR SARSCoV2 OR SARS CoV 2 OR 2019-nCoV OR 2019 Novel Coronavirus OR Coronavirus Disease 2019 OR Coronavirus Disease-19 OR SARS Coronavirus 2) AND TOPIC: (mental wellbeing) AND TOPIC: (Nurse OR Nurses OR Nursing Personnel OR Registered Nurses OR Nursing Staff)<br>#3 TOPIC: (COVID OR Covid OR COVID-19 OR COVID 19 OR COVID19 OR severe acute respiratory syndrome OR coronavirus 2 OR SARS-CoV-2 OR SARSCoV2 OR SARS CoV 2 OR 2019-nCoV OR 2019 Novel Coronavirus OR Coronavirus Disease 2019 OR Coronavirus Disease-19 OR SARS Coronavirus 2) AND TOPIC: (mental health disorders) AND TOPIC: (Nurse OR Nurses OR Nursing Personnel OR Registered Nurses OR Nursing Staff)<br>#4 TOPIC: (COVID OR Covid OR COVID-19 OR COVID 19 OR COVID19 OR severe acute respiratory syndrome OR coronavirus 2 OR SARS-CoV-2 OR SARSCoV2 OR SARS CoV 2 OR 2019-nCoV OR 2019 Novel Coronavirus OR Coronavirus Disease 2019 OR Coronavirus Disease-19 OR SARS Coronavirus 2) AND TOPIC: (depression) AND TOPIC: (Nurse OR Nurses OR Nursing Personnel OR Registered Nurses OR Nursing Staff)<br>#5 TOPIC: (COVID OR Covid OR COVID-19 OR COVID 19 OR COVID19 OR severe acute respiratory syndrome OR coronavirus 2 OR SARS-CoV-2 OR SARSCoV2 OR SARS CoV 2 OR 2019-nCoV OR 2019 Novel Coronavirus OR Coronavirus Disease 2019 OR Coronavirus Disease-19 OR SARS Coronavirus 2) AND TOPIC: (anxiety) AND TOPIC: (Nurse OR Nurses OR Nursing Personnel OR Registered Nurses OR Nursing Staff)<br>#6 TOPIC: (COVID OR Covid OR COVID-19 OR COVID 19 OR COVID19 OR severe acute respiratory syndrome OR coronavirus 2 OR SARS-CoV-2 OR SARSCoV2 OR SARS CoV 2 OR 2019-nCoV OR 2019 Novel Coronavirus OR Coronavirus Disease 2019 OR Coronavirus Disease-19 OR SARS Coronavirus 2) AND TOPIC: (stress disorder OR stress) AND TOPIC: (Nurse OR Nurses OR Nursing Personnel OR Registered Nurses OR Nursing Staff)<br>#7 TOPIC: (COVID OR Covid OR COVID-19 OR COVID 19 OR COVID19 OR severe acute respiratory syndrome OR coronavirus 2 OR SARS-CoV-2 OR SARSCoV2 OR SARS CoV 2 OR 2019-nCoV OR 2019 Novel Coronavirus OR Coronavirus Disease 2019 OR Coronavirus Disease-19 OR SARS Coronavirus 2) AND TOPIC: (psychological distress) AND TOPIC: (Nurse OR Nurses OR Nursing Personnel OR Registered Nurses OR Nursing Staff)<br>#8 TOPIC: (COVID OR Covid OR COVID-19 OR COVID 19 OR COVID19 OR severe acute respiratory syndrome OR coronavirus 2 OR SARS-CoV-2 OR SARSCoV2 OR SARS CoV 2 OR 2019-nCoV OR 2019 Novel Coronavirus OR Coronavirus Disease 2019 OR Coronavirus Disease-19 OR SARS Coronavirus 2) AND TOPIC: (post-traumatic stress OR PTSD) AND TOPIC: (Nurse OR Nurses OR Nursing Personnel OR Registered Nurses OR Nursing Staff)

**Scopus**	#1 (TITLE-ABS-KEY (covid* OR severe AND acute AND respiratory AND syndrome OR coronavirus OR sars-cov-2) AND TITLE-ABS-KEY (mental AND health) AND TITLE-ABS-KEY (nurse* OR nursing*)) AND PUBYEAR > 2019<br>#2 (TITLE-ABS-KEY (covid* OR severe AND acute AND respiratory AND syndrome OR coronavirus OR sars-cov-2) AND TITLE-ABS-KEY (mental AND wellbeing) AND TITLE-ABS-KEY (nurse* OR nursing*)) AND PUBYEAR > 2019<br>#3 (TITLE-ABS-KEY (covid* OR severe AND acute AND respiratory AND syndrome OR coronavirus OR sars-cov-2) AND TITLE-ABS-KEY (mental AND health AND disorders) AND TITLE-ABS-KEY (nurse* OR nursing*)) AND PUBYEAR > 2019<br>#4 (TITLE-ABS-KEY (covid* OR severe AND acute AND respiratory AND syndrome OR coronavirus OR sars-cov-2) AND TITLE-ABS-KEY (depression) AND TITLE-ABS-KEY (nurse* OR nursing*)) AND PUBYEAR > 2019<br>#5 (TITLE-ABS-KEY (covid* OR severe AND acute AND respiratory AND syndrome OR coronavirus OR sars-cov-2) AND TITLE-ABS-KEY (anxiety) AND TITLE-ABS-KEY (nurse* OR nursing*)) AND PUBYEAR > 2019<br>#6 (TITLE-ABS-KEY (covid* OR severe AND acute AND respiratory AND syndrome OR coronavirus OR sars-cov-2) AND TITLE-ABS-KEY (stress AND disorder OR stress) AND TITLE-ABS-KEY (nurse* OR nursing*)) AND PUBYEAR > 2019<br>#7 (TITLE-ABS-KEY (covid* OR severe AND acute AND respiratory AND syndrome OR coronavirus OR sars-cov-2) AND TITLE-ABS-KEY (psychological AND distress) AND TITLE-ABS-KEY (nurse* OR nursing*)) AND PUBYEAR > 2019<br>#8 (TITLE-ABS-KEY (covid* OR severe AND acute AND respiratory AND syndrome OR coronavirus OR sars-cov-2) AND TITLE-ABS-KEY (post-traumatic AND stress OR ptsd) AND TITLE-ABS-KEY (nurse* OR nursing*)) AND PUBYEAR > 2019

Table S3. Quality assessment result of observational studies (*n* = 23) using the questioner according The Agency for Healthcare Research and Quality (AHRQ).

Lp.	First author (year)	The 11-item AHRQ checklist											Total	Quality of study
		1	2	3	4	5	6	7	8	9	10	11		
1.	Abu-Snieneh (2021)	Y	Y	Y	Y	N	N	N/A	N	N	Y	N	5	Moderate
2.	An et al. (2020)	Y	Y	Y	N	N	N	N	N	N	Y	N	4	Moderate
3.	Cai et al. (2020)	Y	Y	Y	Y	N	N	Y	N	Y	Y	N	7	Moderate
4.	Han et al. (2020)	Y	N	Y	Y	N	N/A	Y	N	N	Y	N	5	Moderate
5.	Hong et al. (2021)	Y	N	Y	N	N	N/A	N	N	Y	Y	N	4	Moderate
6.	Hu et al. (2020)	Y	N	Y	Y	N	N	N	N	N	Y	N	5	Moderate
7.	Jiang et al. (2020)	Y	N	Y	N	N	N	N	N	N	Y	N	3	Low
8.	Labrague et al. (2021)	Y	Y	N	N	N	N	N/A	N	N	Y	N	3	Low
9.	Labrague et al. (2020)	Y	Y	Y	N	N	N	N/A	N	N	Y	N	4	Moderate
10.	Li et al. (2020)	Y	N	Y	N	N	N	N/A	N	N	Y	N	3	Low
11.	Pang et al. (2021)	Y	Y	Y	N	N	N	N/A	N	N	Y	N	4	Moderate
12.	Pouralizadeh et al. (2020)	Y	N	Y	N	N	N	N	N	N	Y	N	3	Low
13.	Tu et al. (2020)	Y	Y	Y	N	N	N	N	N	N	Y	N	4	Moderate
14.	Wang et al. (2021)	Y	Y	Y	Y	Y	N	N	N	N	Y	N	6	Moderate
15.	Xiong et al. (2020)	Y	Y	Y	Y	N	N	N	N	N	Y	N	5	Moderate
16.	Yurtseven et al. (2021)	Y	N	Y	N	N	N	N	N	N	Y	N	3	Low
17.	Zheng et al. (2021)	Y	N	Y	Y	N	N	N	N	N	Y	N	4	Moderate
18.	Roberts et al. (2021)	Y	N	Y	N	N	N	N	N	N	Y	N	3	Low
19.	Dal'Bosco et al. (2020)	Y	Y	Y	N	N	N	Y	N	N	Y	N	5	Moderate
20.	Arnetz et al. (2020)	Y	N	Y	N	N	N	N	N	N	Y	N	3	Low
21.	Havaei et al. (2021)	Y	N	Y	N	N	N	N	N	N	Y	N	3	Low
22	Kim et al. (2021)	Y	Y	Y	N	N	N	Y	N	N	Y	N	5	Moderate
23.	Mekonen et al. (2021)	Y	Y	Y	N	N	Y	Y	N	N	Y	N	6	Moderate

Legend: 1: Define the source of information; 2: List inclusion and exclusion criteria; 3: Indicate time period used for identifying patients; 4: Indicate whether or not subjects were consecutive; 5: Whether evaluator covered up other aspects of the subject; 6: Assessments for quality assurance purposes; 7: Explain any patient exclusions from analysis; 8: Describe how confounding was assessed; 9: Explain how missing data were handled; 10: Summarize patient response rates; 11: Clarify percentage of incomplete

Abbreviations: Y: Yes; N: No; N/A: not assessed

Quality of study: low (0-3 yes), moderate (4-7 yes) and high (8-11 yes).
To sum up: 8: Low; 15: Moderate

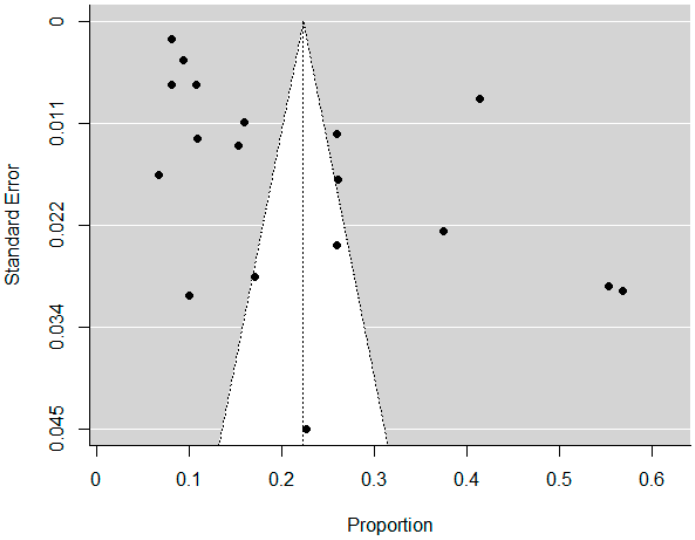

Figure S1. Funnel plot for meta-analysis of the prevalence of depression.

A. B.

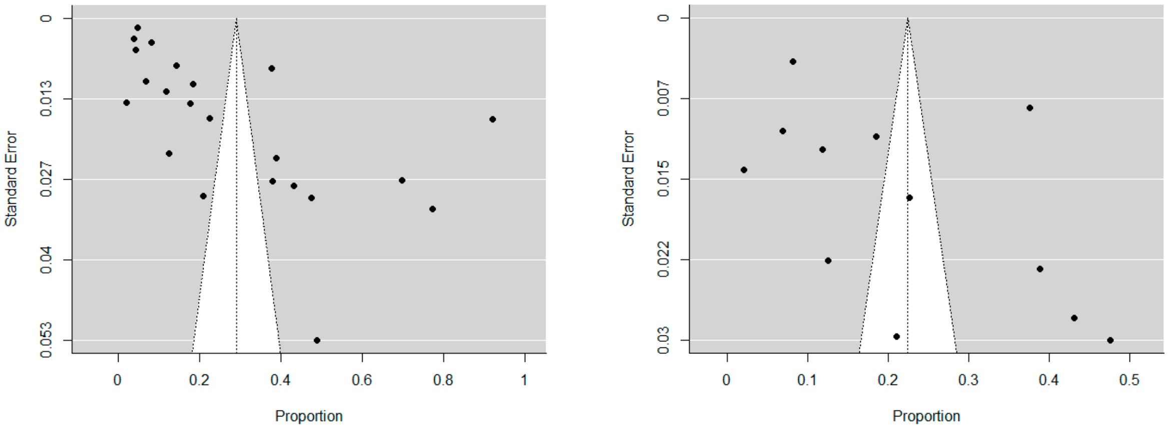

Figure S2. Funnel plot for meta-analysis of the prevalence of depression. A: for all tools; B: only for GAD-7.
